# Optimizing open data to support one health: best practices to ensure interoperability of genomic data from bacterial pathogens

**DOI:** 10.1186/s42522-020-00026-3

**Published:** 2020-10-19

**Authors:** Ruth E. Timme, William J. Wolfgang, Maria Balkey, Sai Laxmi Gubbala Venkata, Robyn Randolph, Marc Allard, Errol Strain

**Affiliations:** 1 U.S. Food and Drug Administration, Center for Food Safety and Applied Nutrition, 5001 Campus Drive, College Park, MD 20740 USA; 2 New York State Department of Health, Wadsworth Center, Albany, New York USA; 3 Association of Public Health Laboratories, Silver Spring, MD USA; 4 U.S. Food and Drug Administration, Center for Veterinary Medicine, Laurel, MD USA

**Keywords:** Genomic epidemiology, GenomeTrakr, Microbial pathogen surveillance, NCBI submission, Whole genome sequencing, QA/QC, One health

## Abstract

The holistic approach of One Health, which sees human, animal, plant, and environmental health as a unit, rather than discrete parts, requires not only interdisciplinary cooperation, but standardized methods for communicating and archiving data, enabling participants to easily share what they have learned and allow others to build upon their findings. Ongoing work by NCBI and the GenomeTrakr project illustrates how open data platforms can help meet the needs of federal and state regulators, public health laboratories, departments of agriculture, and universities. Here we describe how microbial pathogen surveillance can be transformed by having an open access database along with Best Practices for contributors to follow. First, we describe the open pathogen surveillance framework, hosted on the NCBI platform. We cover the current community standards for WGS quality, provide an SOP for assessing your own sequence quality and recommend QC thresholds for all submitters to follow. We then provide an overview of NCBI data submission along with step by step details. And finally, we provide curation guidance and an SOP for keeping your public data current within the database. These Best Practices can be models for other open data projects, thereby advancing the One Health goals of Findable, Accessible, Interoperable and Re-usable (FAIR) data.

## Background

The One Health perspective, which sees human, animal, plant, and environmental health as a unit, rather than discrete parts, requires not only interdisciplinary cooperation, but standardized methods for communicating and archiving data, so researchers can easily share what they have learned and allow others to build upon their findings. Two developments have made a great difference in our ability to support these requirements. First, the advent of whole genome sequencing (WGS) made it possible to establish genomic DNA as a standard data type and increase the resolution possible between isolates, dramatically changing how surveillance data for human pathogens could be stored, shared, and analyzed [1]. Second, storing and sharing genomic pathogen data and surveillance analyses as “open data” [2] has enabled a truly open vision for all global pathogen surveillance, as shown by the success of the open foodborne pathogen surveillance model in the United States [2–4] and in partnering countries, such as the United Kingdom [5], Australia [6, 7], Mexico [8, 9], and Canada [10, 11]. Newer open surveillance efforts for health care acquired illness (HAI) [10–12] and viral diseases [13, 14] are on a similar trajectory for success. An additional benefit of submitting pathogen genomes to public databases in real-time is earlier collaboration around emerging threats, such the COVID-19 pandemic [15] or MCR-1/colistin resistance [16].

While this wide array of public data represents the admirable work of many research teams and their particular areas of interest, it also demonstrates how enthusiastic adoption of technologies can pose challenges for the very database standardization necessary to make these systems useful. As more and more data are collected, differences in methods for data description, analysis, storage, and access could eventually silo our efforts, even within the same pathogen surveillance community. Yet the One Health vision demands that we create systems that can integrate knowledge across species, sources, contributors, and analyses. One of the best ways to honor the hard work and ingenuity which developed these resources would be to ensure such silos do not develop. Instead, we could build upon the data analysis standards recommended by PHA4GE [17] and from existing Best Practices, such as those we describe below. Together we can establish common methods for reliably storing, retrieving, and genomic data for pathogen surveillance.

Many researchers now host their genomic data and primary analyses publicly at the United States National Center for Biotechnology Information (NCBI), which provides a web-accessible view of their databases and supports seamless collaborations across agencies and political borders. Once a day, NCBI uses the International Nucleotide Sequence Database (INSDC) to synchronize with two other important nucleotide databases, European Molecular Biology Laboratory’s European Bioinformatics Institute (EMBL-EBI) and the DNA Databank of Japan (DDBJ) [18] resulting in a truly international database of nucleotide data.

As of March 2020, the genomes of thirty-two important pathogens (31 microbes and one yeast) under active surveillance by public health laboratories and hospitals are now stored at NCBI and are easily available through NCBI Pathogen Detection (NCBI-PD). These genomes, their associated metadata, and automated analysis results can all be accessed through the NCBI-PD browser at https://www.ncbi.nlm.nih.gov/projects/pathogens. First released in 2016, the NCBI PD browser provided the first public analysis portal for bacterial genomic surveillance data in the world [19]. This centralized resource has facilitated collaborations across US agencies, academic partners, non-PulseNet public health laboratories, and international contributors all of which submit and utilize the analysis results for making routine public health decisions. Each day, the NCBI-PD integrates its archived clusters with newly submitted genomes, then computes updated phylogenies for clusters of closely related genomes, which can provide insights about past or ongoing disease outbreaks. These results are available both to the contributing public health labs and to the general public. In addition to phylogenetic clustering, NCBI-PD now screens every bacterial genome for genes associated with antibiotic resistance (AMR) [20], stress response, and virulence, which allows surveillance of specific genes in circulating pathogens by groups such as the National Antimicrobial Resistance Monitoring System (NARMS).

**Fig. 1:**
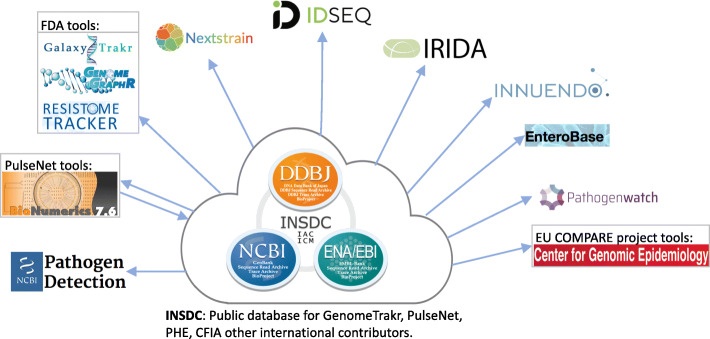
INSDC hub showing how genomic data in public databases get analyzed by many different software platforms, for different purposes. Included in this figure are most genomic epidemiology-related open source analysis platforms available in March of 2020, and one private software tool, BioNumerics. BioNumerics is also the only platform with submission capability

Since its inception in 2016, the NCBI-PD database and surveillance platform has grown in size and importance to public health. Having this central, public resource focused the community around a common set of tools and standards, rather than spending resources creating new tools for each individual lab. Collaborative efforts to improve NCBI-PD have gone beyond the original group of foodborne pathogens to offer real-time clustering of many other pathogens, including *Mycobacterium tuberculosis*, several HIA, and one yeast, *Candida auris*. As the value of having a shared, public resource for genomic surveillance data became obvious, other groups began developing new tools and platforms that utilized NCBI-PD as a common underlying database (Fig. 1). Demand grew for older resources such as BioNumerics (Applied Maths, Sint-Martens-Latem, Belgium), used by the PulseNet community [21], to allow users to submit their WGS data to NCBI, as well as the capability to download NCBI data into BioNumerics to perform local, customized analyses. As a result of building interoperable systems, researchers can now use public data from the INSDC in conjunction with private data from individual or industry labs, using platforms such as Integrated Rapid Infectious Disease Analysis (IRIDA) [22], INNUENDO [23], PathogenWatch [24] NextStrain [13], IDseq [25], and CGE Evergreen [26]. And finally, for FDA-specific missions, open source tools to support genomic epidemiology (GalaxyTrakr) [27], AMR surveillance (Resistome Tracker) [28], and risk assessment (GenomeGraphR) [29] have been created.

Connecting data across species and locations is an essential part of One Health. When a physician, veterinarian, or public health official identifies a case of bacterial or viral illness, they must be able to determine how that datapoint may fit with reports from around the world; that depends on whether the genomic data from the likely pathogen can be analyzed, archived, and made accessible to others. However, accessibility is not simply being able to download genomic sequences economically. It also means storing that sequence with standardized attributes (metadata) that allow important environmental and chain of custody connections to be made. By ensuring that central data sources, such as INSDC and NCBI, are stocked with sequence data and associated metadata submitted using standardized attributes and standard templates, scientists can promote interoperability across multiple platforms and analysis types. These actions can allow a true One Heath resource to emerge: pathogens submitted by different stakeholders from different sources (human clinicals, animals, food, and environmental sources) can all be combined and analyzed for different purposes across different analytic platforms.

Over the last 8 years, laboratories in the FDA’s pioneering GenomeTrakr network [2] have collaborated to build the underlying open-access archive of genomes collected from non-human sources, totaling ~100 K isolates as of July 2020. Sources of these routine surveillance isolates include food (domestic and imported), food production facilities, farms, watershed sampling, and animals from farm, veterinary, and wildlife sources. The resulting public data (genomes and associated metadata), hosted within the NCBI Pathogen Detection, are comprehensive enough to bridge multiple needs (defining outbreaks, identifying their sources, tracking AMR, and primary research), and yet custom NCBI tools and 3rd party analysis platforms meet the unique needs of specific users. As founders with extensive experience and in-depth knowledge of this massive data collection effort, we are pleased to share our Best Practice guidelines here.

## Main text

### Purpose of this document

Our goal for this best practices document is to provide an easy, direct path for any laboratory in the world to participate in a global pathogen surveillance effort. Increasing the number labs able to contribute to NCBI not only democratizes the ability of laboratories to connect their data with others around the world, but also increases the likelihood that the database will capture the range of real-world pathogen diversity. The majority of genomes now available in the NCBI-PD were submitted by two stakeholder groups: 1) national-level teams surveilling foodborne pathogens, including GenomeTrakr [2], PulseNet [4, 21], NARMS [30], and Public Health England [31], among others; and 2) teams surveilling HAI, primarily Brigham and Women’s Hospital [10] and Public Health England [31]. Prior to 2019, most of these submissions were brokered through the large networks, such as GenomeTrakr or PulseNet, rather than from individual laboratories collecting the primary data. With the release of this guidance we are removing GenomeTrakr from the role of being a data broker for our laboratories. In addition, we are taking a step further in making this document broad enough to be relevant for anyone (academics, industry, non-governmental organizations, non-US ministries of health, etc.) interested in starting or contributing to an existing genomic epidemiology effort to have the tools and guidance readily available. The analytical framework has already been established for the 32 pathogens (31 bacteria and one yeast) listed on the NCBI-PD homepage, however communities can request new pathogens be added by submitting a request to NCBI.

Thus, the remainder of this document will describe the NCBI community standards for data collection and provide guidelines for 1) establishing new surveillance projects at NCBI, 2) assessing the quality of your sequence data, 3) submitting raw sequence data and associated isolate metadata to NCBI, and 4) instructions for curating your data and cluster results within specified databases at NCBI. Although the protocols included here were initially developed by the GenomeTrakr team for submitting bacterial, foodborne pathogen isolates collected from non-human sources, these guidelines are written for *any* laboratory that has the following items in place:

- Pure-culture isolates of pathogens (or the ability to amplify a target organism’s entire genome from a swab or sample),
- WGS data of these isolates from Illumina-based sequencing platforms (Miseq, NextSeq, HiSeq, iSeq) that you are willing to share publicly (non-Illumina data can be submitted as assemblies to GenBank),
- Data submission protocols tailored for the NCBI database, although Best Practices apply for INSDC partners (EMBL-EBI and DDBJ) or other databases such as GISAID [27],
- QC Data that meets or exceeds standard QC thresholds (see Table 1),
- Minimum standard metadata ready for submission (see Table 2),
- Submission should include contact information so that agencies or individuals can request additional information, metadata, and/or the isolate, and,
- Process identified to curate your submitted genomes and metadata, keeping them updated, responding to requests, and/or correcting your submissions.

**Table 1:** Quality control threshold guidelines for enterica pathogens collected for GenomeTrakr

Quality metric	*Salmonella enterica*	*Listeria monocytogenes*	*Escherichia coli*	*Shigella sp.*	*Campylobacter jejuni*	*Vibrio parahaemolyticus*
Average read quality Q score for R1 and R2	> = 30	> = 30	> = 30	> = 30	> = 30	> = 30
Average coverage	> = 30X	> = 20X	> = 40X	> = 40X	> = 20X	> = 40X
De novo assembly: Seq. length (Mbp)	~ 4.3–5.2	~ 2.7–3.2	~ 4.5–5.9	~ 4.0–5.0	~ 1.5–1.9	~ 4.8–5.5
De novo assembly: no. contigs	<=300	<=300	<=500	<=650	<=300	<=300

**Table 2:** The minimum set of metadata fields recommended by GenomeTrakr for BioSample submission of bacterial pathogens. Consult the “Populating the NCBI Pathogen metadata template protocol” [32] for expanded, up-to-date guidance

Required fields	Description
strain	This is the authoritative ID used within NCBI Pathogen Detection and for the PulseNet/GenomeTrakr networks. Although the Strain ID can have any format, we suggest that it be unique, concise, and consistent within your laboratory (e.g. CFSAN123456). There are downstream advantages to the name being entirely alpha-numeric, so avoid special characters if possible.
sample_name	Sample Name is another unique identifier for the pure culture isolate and required by NCBI for BioSample submission (it cannot be left blank). It can have any format, but we suggest that it be the same as the strain name or contain another identifier important to the isolate or submitting laboratory. NCBI validates this attribute for uniqueness, so you cannot use “missing, or “not collected”. This identifier is NOT available in NCBI-PD.
organism	The organism name should include the most descriptive information you have at time of submission, adhering to proper nomenclature in NCBI taxonomy database: https://www.ncbi.nlm.nih.gov/Taxonomy/Browser. Check spelling carefully!
collected_by	Name of laboratory that sequenced the isolate (or institute that collected the sample). Abbreviations are ok if they are well-known in the community (e.g. FDA or CDC).
attribute_package	This field provides the pathogen type (or “isolation type”). Allowed values are “Pathogen.cl” (for human clinical pathogens) or “Pathogen.env” (for environmental, food, or animal clinical isolates). The value provided in this field drives validation of other fields and cannot be left blank.
collection_date	Date of sampling in ISO 8601 standard: “YYYY-mm-dd”, “YYYY-mm” or “YYYY” (e.g., 1990–10–30, 1990–10, or 1990).
geo_loc_name	Geographical origin of the sample using controlled vocabulary: http://www.insdc.org/documents/country-qualifier-vocabulary. Use a colon to separate the country or ocean from more detailed information about the location, e.g., “Canada: Vancouver”. Country and state are required for GenomeTrakr isolates from the US, e.g. “USA: CA”.
isolation_source	Describes the physical, environmental and/or local geographical sample from which the organism was derived. Avoid generic terms such as patient isolate, sample, food, surface, clinical, product, source, environment.
host	^a^For Pathogen.cl only: “*Homo sapiens*” if clinical isolate.
host_disease	^a^For Pathogen.cl only: Name of relevant disease, e.g., Salmonella gastroenteritis. This field must use controlled vocabulary provided at: http://bioportal.bioontology.org/ontologies/1009 or http://www.ncbi.nlm.nih.gov/mesh. Label this field “not collected” if unknown for clinical isolates. Leave blank for all Pathogen.env isolates.
bioproject_accession	The accession number of the BioProject(s) to which the BioSample belongs (PRJNAxxxxxx).
lat_lon	Provide latitude and longitude to support “geo_loc_name”. This field is required to be populated by NCBI. However, if this level of detail is not available, GenomeTrakr recommends including “missing” or “not collected” here.

^a^ “For Pathogen.cl only”: These fields are mandatory ONLY if isolate is from a human clinical sample. If isolate was collected from food/water/env or animal sources, these fields should be left blank

### The importance of standardized metadata

Ensuring that your laboratory can provide the minimum set of metadata should be done as the project is getting started, BEFORE any sequencing starts or submissions are prepared. In order for pathogen surveillance to be successful, we need standard metadata for each pure culture isolate, especially when there are numerous independent laboratories collaborating in the effort. As an example, the minimum metadata fields for GenomeTrakr are as follows:

- laboratory name holding the isolate,
- unique strain ID,
- isolation type (human source vs. non-human source),
- collection date (year minimum),
- isolation source (e.g. type of food, animal, or description of environment),
- and location (Country *and* state if in US).

**Fig. 2:**
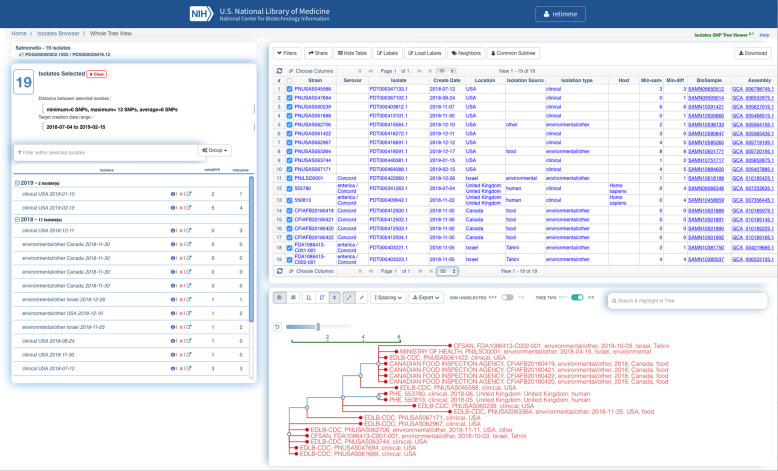
Screen shot of a cluster within the NCBI-PD browser showing harmonized metadata submissions across five different submitting laboratories (PulseNet, GenomeTrakr, Public Health England, Israel Ministry of Health, and CA Food Inspection Agency). URL: https://www.ncbi.nlm.nih.gov/Structure/tree/#!/tree/Salmonella/PDG000000002.1922/PDS000025876.12?treelabel=sra_center,strain,epi_type,collection_date,geo_loc_name,isolation_source

It is equally important to ensure that each piece of metadata gets submitted to the correct metadata attribute (or field) within the package (Table 2), or the information will not get labeled properly and therefore will not be available to those interpreting the results. The INSDC, in collaboration with the Global Microbial Identifier (GMI) (https://www.globalmicrobialidentifier.org), recommends using the Pathogen metadata template for pathogen surveillance submissions: (NCBI: https://submit.ncbi.nlm.nih.gov/biosample/template/?package=Pathogen.combined.1.0&action=definition and EMBL-EBI: https://www.ebi.ac.uk/ena/submit/pathogen-data). Following the GenomeTrakr metadata guidelines described in the “Submitting metadata” section will enable your data to be Findable, Accessible, Interoperable, and Reusable, also known as meeting FAIR standards [33]. This will also allow your laboratory’s submissions to get properly analyzed, integrated, and labeled on the resulting tree clusters within the NCBI-PD browser (Fig. 2).

### Quality control standards: how to QC data

Quality control (QC) thresholds help ensure the interoperability, accuracy and usefulness of NCBI-PD resources. It is important that contributors only upload material which meets quality control (QC) thresholds for both the metadata and the underlying sequence data. For example, data from an environmental isolate that fails to include descriptive metadata about isolation source, location, or date of collection is of little use in helping inform epidemiologists about potential exposures during an outbreak. Although it is possible to cluster such data, it cannot provide guidance for the investigation. Similarly, if poor quality sequence data is submitted for clinical isolates, those cannot be reliably clustered, resulting in missed opportunities for early detection of an outbreak. Guidance for metadata QC will be given later in this document.

Quality control thresholds recommended for sequence data from bacterial foodborne pathogens, based on current Illumina sequencing technology, are provided in Table 1. We recognize that these thresholds may need to be revised in the future, since continuing improvements to sequencing technologies, as longer read lengths and lower error rates, should lead to lower coverage requirements and better de novo assemblies (thresholds kept current in the “Assessing Sequence Quality in GalaxyTrakr” protocol [34]. Data passing these QC thresholds can generally be considered fit for purpose for identifying clusters of isolates involved in foodborne outbreaks, as well as for identifying many antimicrobial resistance and virulence/pathogenicity elements. These levels of quality also support the cgMLST approaches currently used by members of CDC PulseNet.

**Fig. 3:**
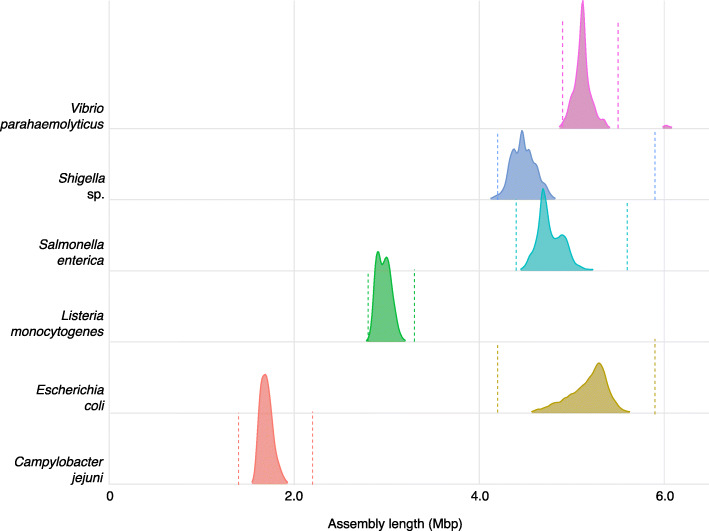
Density plot showing the distribution of genome lengths for a random sample of isolates with Illumina sequence data available from NCBI Pathogen Detection portal (*n* = 10,000 for all species except *V . paramaemolyticus* where *n* = 1414 due to smaller number of samples). Sequences were assembled using SKESA 2.2 and the bars indicate ±3 standard deviations from the mean. Mbp = mega base pairs

**Fig. 4:**
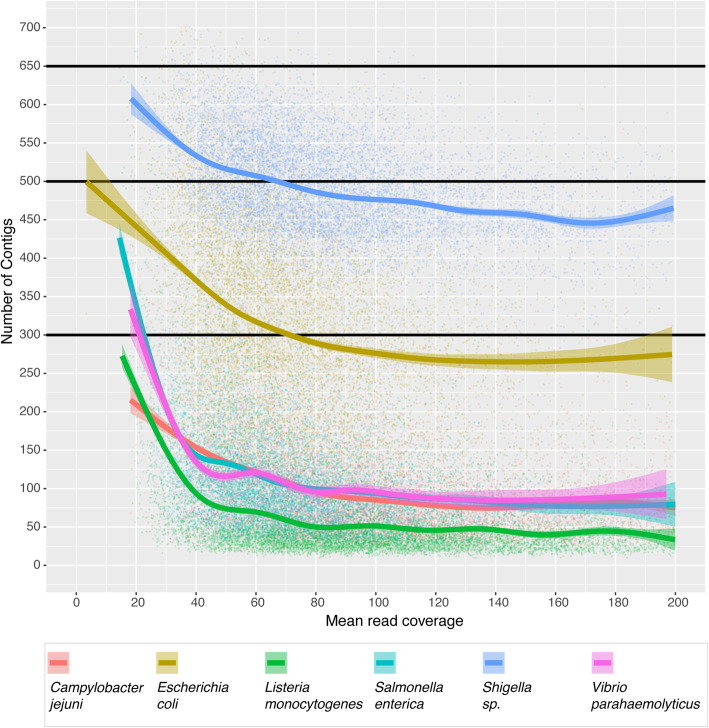
Plot of mean coverage (as reported by SKESA v. 2.2) vs number of contigs for a random sample of isolates with Illumina sequence data available from NCBI Pathogen Detection portal (n = 10,000 for all species except *V. parahaemolyticus* where n = 1414 due to smaller number of samples). The smoothed line was generated using generalized additive smoothing in R. Assembly quality, as measured by a decrease in the number of contigs, generally increases with increasing coverage

The values in Table 1 are empirically derived from a large sample of isolates at NCBI-PD that were shotgun sequenced using Illumina technology. For each of the *Salmonella enterica*, *Listeria monocytogenes*, *Escherichia coli*, *Shigella sp.*, and *Campylobacter jejuni* databases 10,000 random isolates were selected on January 6th, 2020 from each respective NCBI-PD isolate table. On Jan 9th, 2020 1414 of the available *Vibrio parahaemolyticus* isolates were selected, reflecting its smaller database. A summary of these data (51,414 isolates) shows the range of de novo assembly lengths, defined as the sum of the length of all contigs, for six pathogens relevant for foodborne outbreaks (Fig. 3 and Additional file 1). These results highlight the difficulty in establishing narrow guidelines for foodborne bacteria sequencing metrics as the assembly length can be highly variable as observed in *E. coli*, or multi-modal as in *S. enterica* and *L. monocytogenes*. Increasing coverage does appear to improve the quality of de novo assemblies, as measured by number of contigs, but that the rate of improvement slows once coverage exceeds about 40X (Fig. 4). Importantly, as genomes for different pathogens vary in size and complexity, the coverage needed to obtain a good *L. monocytogenes* or *C. jejuni* assembly does appear to be less than for larger, more complex genomes like *Shigella* and *E. coli*. GenomeTrakr recommendations for coverage represent a compromise between sequencing cost and data quality (Table 1 and [29]). As isolates are typically barcoded and multiplexed, often including isolates from multiple different surveillance efforts on the same sequencing run (e.g. a single MiSeq run could have isolates intended for GenomeTrakr, PulseNet, tuberculosis surveillance, and/or HAI), we strongly recommend performing QC on the *entire* run, ensuring that sample swaps, misidentifications, and/or contamination can be properly identified.

If a laboratory and research group does not have bioinformatic capacity, it can be challenging to assess if samples meet relevant QC thresholds. Even for teams that do have bioinformatics resources, it can take significant time to develop the kinds of reports and dashboards that allow quick decisions about whether a sample needs to be re-analyzed or if there are larger issues with a sequencing run. We have removed this hurdle by providing a custom, cloud-based workflow on the Galaxy platform [35], called “MicroRunQC”, that generates a general QC report appropriate for most microbial pathogens, summarizing sequence quality, coverage, assembly quality, and sequence typing. Laboratorians can upload their FASTQ sequence data to our custom Galaxy instance, called GalaxyTrakr (https://galaxytrakr.org), and run the MicroRunQC workflow [34], producing a QC report in less than an hour for a typical sequencing run of 24–36 isolates. Laboratories should review this QC report to verify that their sequence quality, coverage, and assembly quality thresholds are indeed acceptable for the target organism(s). Sequencing type (ST) results should also be assessed to make sure that the genus/species predictions match the input sample. While this approach will not detect sample swaps between closely related bacterial strains or low levels (< 5%) of contamination, the ST results can identify putative swaps across more divergent strains. Additionally, isolates with multiple ST allele calls (i.e. two or more alleles at one gene) should be further reviewed for evidence of contamination (refer to QC Failures section).

An important feature of the MicroRunQC workflow is that it was built using open-source tools and therefore could be implemented on local bioinformatics systems [36]. As with many of the resources and pipelines used within the GenomeTrakr network, the MicroRunQC workflow is not restricted to the common foodborne bacterial pathogens and can be used to track sequencing metrics on a range of pathogens commonly encountered in public health surveillance such as *Neisseria, Legionella, Mycobacterium*, etc. Extending QC parameters to encompass these and other organisms is an ongoing project, and collaborators are welcome to help establish these.

### Identifying anomalies and performing root cause failure analyses

As more *species* and *sources* of genomic data are brought into the NCBI-PD, the more essential it becomes that we can all rely on the quality control efforts of participating laboratories. Trust is an essential part of One Health. Ideally all bacterial WGS data meet the QC criteria specified by the coordinating sequence network (e.g. GenomeTrakr requirements listed in Table 1), and when that quality is confirmed, the laboratorians can continue with the next steps of submitting the data to NCBI .

However, when QC failures are detected, laboratorians are faced with a handful of decisions, depending on the type of failure identified. We will provide general guidance to the most common errors flagged in the MicroRunQC report. The most damaging type of errors, for One Health surveillance purposes, are those which relate to proper labeling of the samples. Do we have what we expect, based on the provided metadata? These errors include:

1. If the genus or species for one or more samples does not match what is predicted in the MLST results
2. If the genome size is higher or lower than expected – this is potential evidence of sample swap, contamination, or mixed cultures, and
3. Number of contigs much higher than expected – this is potential evidence of contamination, mixed cultures, or poor quality sequence data.

Root cause investigations into these sorts of errors can be as simple as checking sample sheets to confirm that barcodes/indices were used correctly, and reviewing laboratory notebooks for evidence of sample swaps. However, it can also be helpful to use other bioinformatic tools such as serotype predictions, clustering results, metagenomic analysis, and comparing antimicrobial resistance genotypes to phenotypes if the information is available. If errors remain and the provenance of the WGS data still cannot be established, it may be necessary to re-isolate the bacteria from the original samples, holding off data submission until QC issues have been resolved.

Not all errors rise to the level of questioning the labeling or provenance of a sample. Some QC errors affect the usefulness of the data for downstream applications, and result from low read quality, low coverage, or high number of contigs. Samples exhibiting these problems usually provide enough usable sequence data to verify species; however, the sequence quantity is insufficient for downstream analysis. These types of errors can result from problems with library preparation and potentially loading too little or too much DNA onto the sequencing instrument. Typically, these mistakes can be fixed by either re-sequencing DNA libraries or preparing new libraries from genomic DNA. Although NCBI-PD will accept and process data that is of lower quality, decisions about how and when to correct minor errors may depend upon the resources of the originating laboratory and the uniqueness of the specific isolate.

### NCBI data submission overview

**Fig. 5:**
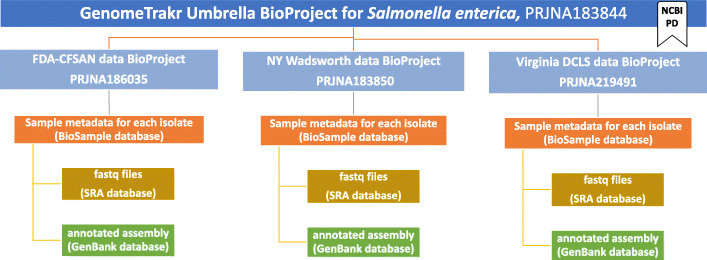
Overview of the database structure at NCBI showing an example *Salmonella* umbrella BioProject with three linked laboratory data BioProjects, each with their own BioSamples and associated sequence data

Before starting your first data submission it is imperative first establish your submission environment at NCBI. For most pure-culture microbial surveillance projects the BioProject structure will be taxonomically focused (Fig. 5); therefore, a BioProject structure will first be established around each pathogen species of interest. Each species-specific BioProject will contain both the isolate metadata (BioSamples) and the sequence data collected from each isolate (raw sequence at SRA and/or annotated assemblies in GenBank). Accompanying this Best Practices is a custom NCBI submission protocol [37] tailored for genomic epidemiology data submission, which represents an expansion of Timme et al.’s submission protocol [38].

#### Creating BioProjects

BioProjects are an organizing tool at NCBI [39, 40] that pull together different kinds of data submitted across multiple NCBI databases. Each BioProject contains a unique URL, providing a home page with a title, description, links to lab websites, publications, funding resources associated with a particular project, along with links to the deposited data. A basic BioProject holds actual sequence data, assemblies, and their associated metadata. An umbrella BioProject is a way to group two or more basic BioProjects together, which is useful for disease surveillance and for looking across the grouped BioProjects in a single view. If specific umbrella BioProjects are intended for disease surveillance on the NCBI-PD, then the project can be “flagged” reflecting this intention. The result is that any sequence data submitted to a flagged BioProject (data or umbrella BioProject) will automatically get processed through the NCBI-PD pipeline. For example, in the GenomeTrakr network there is one flagged umbrella BioProject for each pathogen species under active surveillance at NCBI-PD (e.g. one for *Salmonella, Listeria*, etc.). The *Salmonella* umbrella contains 51 data BioProjects, each associated with a contributing laboratory and each inheriting the umbrella NCBI-PD flag: https://www.ncbi.hlm.nih.gov/bioproject/183844. Individual data BioProjects can also be flagged, enabling flexibility for different types of submitters. Consult the NCBI submission protocol [37] for linking to or establishing new umbrella and data bioprojects. If your laboratory is already a member of an established surveillance network you should first check to see if they would like you to link to their existing umbrella BioProject. For reference, the GenomeTrakr umbrella BioProjects are listed and kept current within this protocol.

#### Submitting metadata

The BioSample database at NCBI is designed to hold the metadata for “samples,” or biological source materials [39], which can be many different things depending on your research. For microbial pathogen surveillance and GenomeTrakr these materials are the pure-culture bacterial isolates. Before collecting sequence data for your isolates, ensure that you can provide the minimum metadata recommended by your coordinating surveillance body. In Table 2, we provide the GenomeTrakr guidance on how to populate the Pathogen metadata package (see Appendix D for expanded guidance), along with a core set of recommended fields that users should populate with contextual data. Step-by-step instructions for submitting isolate metadata to NCBI are given in the “BioSample creation” section of the NCBI submission protocol [37]. If it becomes necessary to update, correct, or retract a Biosample registration, consult the NCBI Data Curation Protocol [41].

#### Submitting sequence data

NCBI comprises separate databases that hold the different types of DNA, RNA, or assembled sequence data [42]. For example, the nucleotide database (often called, simply, “GenBank”) holds annotated DNA or RNA sequence data, complete or draft bacterial genomes, complete chloroplast or mitochondrial genomes, individual gene sequences, and phylogenetic alignment datasets, such as internal transcribed sequence (ITS) datasets. The Sequence Read Archive (SRA) [42, 43] database houses unannotated raw high throughput DNA or RNA sequence reads: for microbial pathogen surveillance these are the Illumina FASTQ file sequences collected from the pure cultured isolates registered in the BioSample database. For each isolate registered in Biosample, there is usually one associated SRA submission (only one SRA submission per isolate is recommended), linked by the run accession (SRR#######). A separate draft or complete genome *can* be submitted to GenBank under the same BioSample accession, but this is not required. NCBI submits the annotated assemblies they create as part of the Pathogen Detection Pipeline to GenBank. A detailed protocol for submitting raw sequence data to SRA is included in the NCBI Submission protocol.

### Data management and curation

#### Establishing a data submission workflow

If new pathogen species or participating laboratories are added to a surveillance effort after the initial BioProject is established and linked to the Pathogen Detection pipeline, it may be useful to create new BioProjects, especially if the labs have multiple projects they need to track separately. A new BioProject Umbrella should be created if a previously unsurveilled organism is added the surveilled pathogens list, or a new surveillance network has been established. It is also reasonable for countries to request a new Umbrella – e.g., Public Health England uses the Umbrella PRJNA248064 to organize all their submissions. After the BioProjects are established, the routine submission workflow for an individual laboratory would only include submission to two databases, BioSample and SRA.

#### Responsibilities of contributors

The transition to genomics and open, public systems for pathogen surveillance brings new roles and responsibilities for scientists working in laboratories and public health professionals who use WGS results to resolve outbreaks and identify sources of contamination. Laboratories must build capacity to perform bioinformatic analysis on genomic data, whether locally or via cloud-based tools (e.g. MicroRunQC in GalaxyTrakr as an example), to assess data quality and to support outbreak response and traceback efforts. For their part, epidemiologists also need to gain some understanding of molecular evolution and phylogenetics so they can effectively integrate genomic findings with traditional data sources. In addition, laboratories need to develop internal processes to ensure that data collection is recorded consistently, that it is accurately submitted, and that both metadata and sequence data in those archives are kept current. This last part is crucial to effective surveillance of public data across organizations and for integration of data from different partners into a One Health Framework. The quality of any shared resource depends on the willingness of contributors to maintain the records they submit.

Maintaining current and updated data is an *extremely* important part of utilizing these data for public health surveillance. Over the course of the sequence data collection (from culturing, through genome sequencing, to internal QC and data submission) it is normal to have a low rate of certain errors, such as sample switches, spelling errors, cut/paste errors, or mis-identified isolates. Although each of the coordinating surveillance network bodies (GenomeTrakr, PulseNet, Vet-LIRN, etc) should work to minimize these errors, each submitting lab must be also diligent about correcting public data as soon as errors are discovered. To facilitate this process, each lab should have a documented curation procedure to ensure data are updated in a timely manner. The task of data curation could be specifically assigned a person or team of people trained for this important task and might involve establishing new, routine communication channels between disparate groups (i.e. sample collection and isolation, sequencing workflow, data analysis, and data submission might all be done by independent teams). Depending on the volume of submissions within a laboratory, the Data Curation workload could become a significant part of a team’s effort. We have outlined the step-by-step process in our NCBI Data Curation protocol [41].

## Conclusion

To enable easy reference, we will keep the protocols and guidelines published with this manuscript current at protocols.io, which will also allow our recommendations to evolve with advances in technology, improvements in metadata interoperability, and expansion of NCBI-PD capabilities. We hope these Best Practices will reduce the high-learning curve experienced by most new submitters to these databases, spurring more laboratories to use the NCBI-PD surveillance tools and participate by submitting sequences and metadata. As more contributors join the effort, these databases will become a richer source of isolates help realize the One Health goal of integrating the understanding of human, animal, and environmental pathogens, along with their sources. A true global surveillance effort fed by hundreds of submitters around the world, all with common standard metadata, quality control, and submission procedures would be groundbreaking for public health, to the advancement of science, and to the overall One Health ideal.

## Supplementary information

**Additional file 1. **SRA accessions for 10,000 random isolates sampled from NCBI Pathogen Detection for *S. enterica*, *L. monocytogenes*, *E. coli, Shigella* sp., *C. jejune*, and *V. parahaemolyticus* (1400 due to smaller database).

## Data Availability

All data analyzed during this study are included in this published article including NCBI accessions listed in Additional file 1. We also published four new protocols, referenced in this manuscript with DOI links to their home at protocols.io (https://www.protocols.io/groups/genometrakr1/publications), and provided a script to install a local analysis workflow, hosted on GitHub (https://github.com/estrain/MicroRunQC).
